# SARS-CoV-2 Serological testing in frontline health workers in Zimbabwe

**DOI:** 10.1371/journal.pntd.0009254

**Published:** 2021-03-31

**Authors:** Simbarashe Rusakaniko, Elopy Nemele Sibanda, Takafira Mduluza, Paradzayi Tagwireyi, Zephaniah Dhlamini, Chiratidzo Ellen Ndhlovu, Precious Chandiwana, Shingirai Chiwambutsa, Rivka May Lim, Fiona Scott, Lindiwe Majele Sibanda, Francisca Mutapi

**Affiliations:** 1 Faculty of Medicine and Health Sciences, University of Zimbabwe, Avondale, Harare, Zimbabwe of Zimbabwe; 2 Asthma Allergy and Immunology Clinic, Twin Palms Medical Centre, Harare, Zimbabwe; 3 Department of Pathology, National University of Science and Technology Medical School, Bulawayo, Zimbabwe; 4 Department of Biotechnology and Biochemistry, University of Zimbabwe, Mount Pleasant, Harare, Zimbabwe; 5 Department of Geography and Environmental Science, Geo-information and Earth Observation Centre, University of Zimbabwe, Mount Pleasant, Harare, Zimbabwe; 6 Institute of Immunology & Infection Research, University of Edinburgh, Ashworth Laboratories, Edinburgh, United Kingdom; 7 NIHR Global Health Research Unit Tackling Infections to Benefit Africa (TIBA), University of Edinburgh, Ashworth Laboratories, Edinburgh, United Kingdom; 8 Iam4BYO-Fighting COVID-19, Castell Court, Bulawayo, Zimbabwe; University of Oxford, UNITED KINGDOM

## Abstract

**Background:**

In order to protect health workers from SARS-CoV-2, there is need to characterise the different types of patient facing health workers. Our first aim was to determine both the infection status and seroprevalence of SARS-CoV-2 in health workers. Our second aim was to evaluate the occupational and demographic predictors of seropositivity to inform the country’s infection prevention and control (IPC) strategy.

**Methods and principal findings:**

We invited 713 staff members at 24 out of 35 health facilities in the City of Bulawayo in Zimbabwe. Compliance to testing was defined as the willingness to uptake COVID-19 testing by answering a questionnaire and providing samples for both antibody testing and PCR testing. SARS-COV-2 antibodies were detected using a rapid diagnostic test kit and SAR-COV-2 infection was determined by real-time (RT)-PCR. Of the 713 participants, 635(89%) consented to answering the questionnaire and providing blood sample for antibody testing while 560 (78.5%) agreed to provide nasopharyngeal swabs for the PCR SARS-CoV-2 testing. Of the 635 people (aged 18–73) providing a blood sample 39.1% reported a history of past COVID-19 symptoms while 14.2% reported having current symptoms of COVID-19. The most-prevalent co-morbidity among this group was hypertension (22.0%) followed by asthma (7.0%) and diabetes (6.0%). The SARS-CoV-2 sero-prevalence was 8.9%. Of the 560 participants tested for SARS-CoV-2 infection, 2 participants (0.36%) were positive for SAR-CoV-2 infection by PCR testing. None of the SARS-CoV-2 antibody positive people were positive for SAR-CoV-2 infection by PCR testing.

**Conclusion and interpretation:**

In addition to clinical staff, several patient-facing health workers were characterised within Zimbabwe’s health system and the seroprevalence data indicated that previous exposure to SAR-CoV-2 had occurred across the full spectrum of patient-facing staff with nurses and nurse aides having the highest seroprevalence. Our results highlight the need for including the various health workers in IPC strategies in health centres to ensure effective biosecurity and biosafety.

## Author summary

The SARS-CoV-2 pandemic has reinforced that health workers are critical for the functioning of a health system and that they need to be protected from infection. To provide biosecurity and biosafety for health workers for SARS-CoV-2 infection prevention and control (IPC), it is critical to identify who the patient-facing health workers are in a specific health system. We therefore conducted our study in Zimbabwe to characterise the different workers coming into contact with patients and their exposure to COVID-19. The health workers were defined by occupation and work station within the health centre. The study showed that over 75.0% of the health workers were female, and that the largest group of patient-facing health workers were nurses who constituted 45.3% of the workforce. The largest group, 43.0% of the workforce, was based in the clinical wards. Sero-reactivity among these health workers was 8.9% with the highest prevalence being in the nurses. Two participants (0.36%) were positive for SARS-CoV-2 infection by PCR testing. None of the SARS-CoV-2 antibody positive people were positive for SARS-CoV-2 infection by PCR testing.

## Introduction

The SARS-CoV-2 pandemic reached the African continent in March 2020 exposing African health systems to an additional infectious disease challenge. Zimbabwe reported its first case of COVID-19 on March 21^st^ 2020. The early COVID-19 cases highlighted the need to strengthen the country’s response to the SARS-CoV-2 pandemic. Biosecurity and Biosafety became a critical aspect of infection prevention and control practices (IPC) in hospitals and clinical facilities with the World Health Organisation issuing guidance on various aspects of IPC [1]. While the recommendations applied globally, it was clear that health systems and the staff working in them differed between continents and countries. Thus, in order to fully implement the IPC guidelines it was critical to know the types and levels of interactions different patient-facing staff had in order to establish an operational COVID-19 definition of a frontline health worker within the context of the African health system. This issue had been discussed by people working within African health systems in different countries e.g. Ghana [2]. We conducted this study to characterise the COVID-19 patient-facing workforce within Zimbabwean health centres. Prior to this study, doctors and nurses were prioritised for the provision of PPE. We determined the seroprevalence of SARS-CoV-2 antibodies in the patient-facing health workers in Zimbabwean health centres in order to generate data pertaining to COVID-19 exposure amongst different patient-facing health workers. This information would inform the country’s IPC strategy to prevent nosocomial infections, community spread of infection by health workers as well as protecting the healthcare workforce [3] as highlighted in other studies [4].

Several studies have already indicated that frontline health workers globally e.g. in Italy by April 3^rd^, 2020, around 10,000 healthcare workers had been infected and 74 had died [5] with more deaths recorded in countries across the globe [6]. On July 23^rd^, the WHO reported about 10% of all COVID-19 cases globally were among health workers and more than 10 000 health workers in the 40 African countries which had reported on COVID-19 infections in health care workers had been infected with COVID-19 [7]. In neighbouring South Africa as of August 2020, 27 000 had been infected and 240 lost their lives to COVID-19 in the line of duty [8]. This highlighted the importance of protecting health workers. Our first aim was thus, to determine both the infection status and seroprevalence of SARS-COV-2 in health workers in the health centres as the epidemic was in the early rising phase, when the country had reported 314 cases of SARS-COV-2. Using the information obtained from characterising the patient facing health workers in terms of work station, job station, demographics and comorbidities that are potential risk factors for SARS-CoV-2 infection and disease as detailed by the CDC [9], together with the seroprevalence data, our second aim was to evaluate the occupational and demographic predictors of seropositivity.

## Methods

### Ethics statement

The study received institutional approval from the University of Edinburgh and Ethical approval from the Medical Research Council of Zimbabwe (MRCZ/A/2602). Permission to conduct the study in the province was obtained from the Provincial Medical Director, the City Director of Health Services and the Heads of the Health Facilities. Prior to enrolment, the study aims and procedures were explained to all participants in English. Written informed consent was obtained from the participants for each stage of the study (questionnaire, antibody testing and PCR testing). Recruitment into the study was voluntary. The study was conducted according to the ethical guidelines and principles of the International Declaration of Helsinki, the Guidelines for Good Clinical Practice in Zimbabwe and the Medical Research Council Ethical Guidelines for Research.

### Study site and period

**Fig 1:**
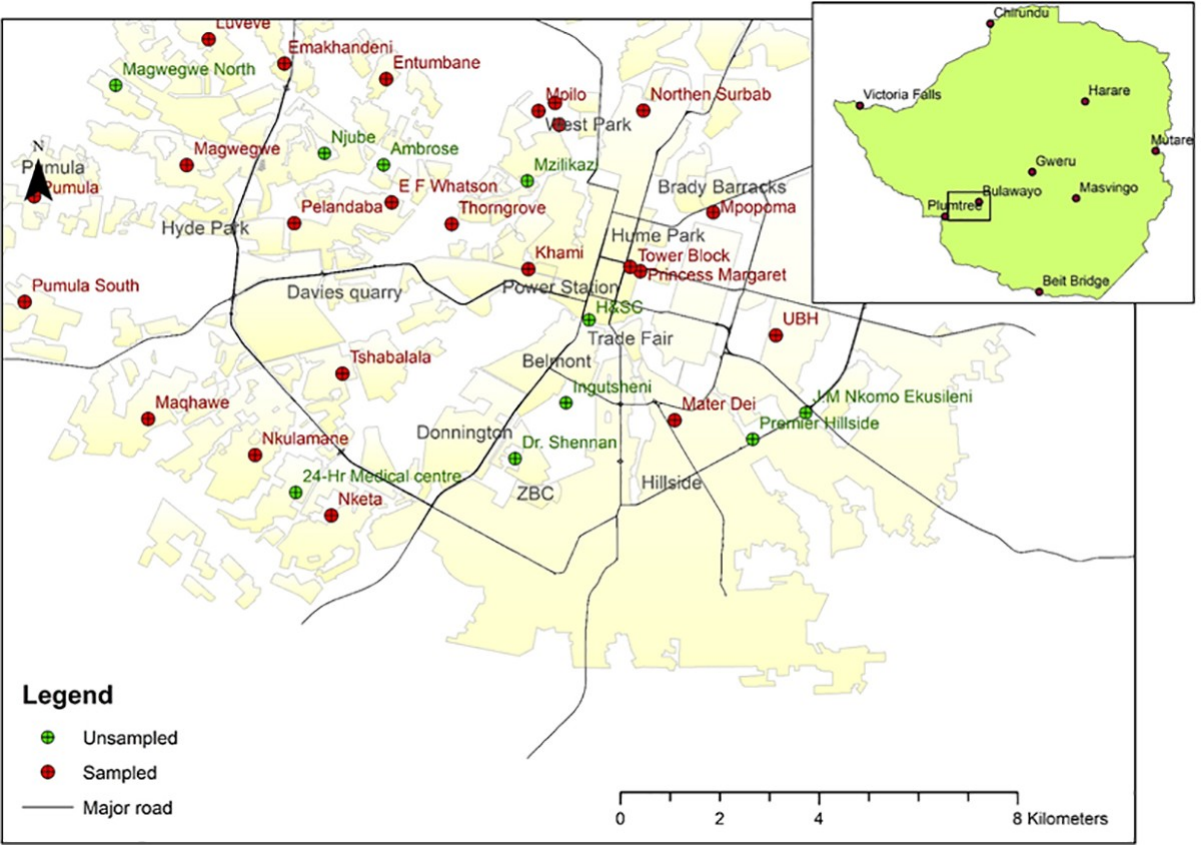
Map showing all health facilities in Bulawayo district and highlighting the 24 sampled health facilities. Baselayer created using ARCGIS https://www.arcgis.com/index.html.

The study was conducted in 24 of the 35 health facilities (private, Council clinics and government hospitals) in the Bulawayo District (20.1457° S, 28.5873° E, and see Fig 1). These health centres were selected for the study because 1) the health workers were working in the designated COVID-19 isolation and reception health facilitates in the region and, 2) they are also the designated centres for travellers from South Africa, during a period when a significant number of Zimbabwe’s cases were from returnees to the country with significantly fewer cases being locally acquired e.g. of the 22 new cases recoded in Zimbabwe on June 22nd, 20 had been returnees from South Africa and 2 were acquired locally.

Of the 24 health facilities sampled, 14 are owned by the Bulawayo City Council, 6 are owned by the Government of Zimbabwe, 3 are privately owned and 1 i.e. Ekuphumuleni, not a health facility but a geriatric nursing home funded by Non-Governmental Organisations. This mixed sampling approach was to allow the study to capture different types of patient-facing worker in different health care settings.

The epidemiological context of this study is as follows; the United Nations Office for the Coordination of Humanitarian Affairs Zimbabwe Situation Report of the 26^th^ of June 2020 indicated that from 20^th^ March to 24^th^ June Zimbabwe recorded 530 COVID-19 confirmed cases and 6 deaths [10]. The study ran from June 9th to June 22^nd^ 2020. The Ministry of Health’s daily COVID-19 reports indicated that on June 9^th^, the country had recorded a total of 314 cases and 4 deaths; 22 cases and 1 death had been reported in Bulawayo. On our last day of sampling, June 22^nd^, the cumulative COVID-19 cases reported in the country was 512 and 6 deaths, with 64 cases reported in Bulawayo and 2 deaths. The WHO AFRO and TIBA COVID-19 situational reports indicate that Zimbabwe’s SARS-CoV-2 epidemic was in the early rising stage during this sampling period.

### Patient and public involvement

The study was conducted as a response to a request by doctors’ groups in Zimbabwe. Prior to the study, the team participant engagement lead arranged webinar series where all co-authors attended and received input and feedback on the study design from participants and representatives of the health centres involved. The team leader in Zimbabwe also hosted a question and answer session on a local radio station for members of the public. Following the incorporation of the feedback into the methodology, an in-person workshop was held in Bulawayo for policy makers from the ministry of health as well as local stakeholders and executive heads of the health centres prior to the commencement of the study. During the course of the study, daily feedback was solicited from the participants on the operational aspects of the study that could be improved for other participants. Post-study a dissemination workshop on lessons learnt was run with the team receiving feedback from the participants. Individual test results were disseminated as per protocol for test results prescribed by the Ministry of Health in Zimbabwe to ensure confidentiality and psychosocial counselling by the qualified members of our team if required. For knowledge uptake and policy guidelines, the policy-relevant findings were presented to the Ministries of Health and Science and Technology in-person prior to the preparation of this manuscript.

### Participant recruitment

Our approach was to recruit every worker who comes into contact with patients on a daily basis, i.e. patient-facing worker who consented at each of the selected 24 health facilities. Thus, all patient-facing workers ranging from security, cleaners, laundry, student nurses, nurses, administrators and doctors were included in the study population. Demographic data provided by the Ministry of Health indicated that the total maximum of 1000 employees and thus, this was the study target population. Prior to recruitment, a webinar was held with heads of the health facilities to give the background, aims and procedures of the study. This was followed by a presentation to potential participants and an official sensitization launch of the study.

### Determining compliance

Compliance to testing was defined in this study as the willingness to uptake COVID-19 testing by answering a questionnaire and providing samples for antibody testing (blood) and PCR testing (nasopharyngeal swabs). A questionnaire with a demographic section, clinical characteristics section and history of exposure section was developed to assess the willingness of the participants to undergo SARS-CoV-2 antibody and PCR testing. The questionnaire (available on: http://tiba-partnership.org/tiba/sites/sbsweb2.bio.ed.ac.uk.tiba/files/protocols/Final_Final_Covid-19%20Questionnaire_27_05_2020%20%281%29.pdf) was developed, validated, pre-tested and modified accordingly prior to use in the study. Through the questionnaire, the participants were asked questions on exposure risks (COVID-19 patient, co-worker, and household contact) and current and previous symptoms and self-reported comorbidities. The questionnaire was administered using the face to face interview approach with enumerators entering the responses on an electronic form on the Itel P33 Plus android smartphones. Data for the whole study including the questionnaire data were captured electronically on android devices using the FieldTask application (Smap Consulting, Niel Penman, VIC, Australia), quality checked and downloaded into the master database every day. Compliance rate was calculated as the percentage of the number of people answering the questionnaire or giving a blood sample for antibody testing or giving a nasal swab for PCR testing depending on the variable being measured out of the total number of consenting participants.

### Sample collection and antibody testing

All sample collection was done by a clinician. For the antibody testing, a 5 ml venous blood sample was collected from each participant, separated into serum and 40 ul of the serum was used for detecting the presence of SARS-CoV-2 antibodies using a rapid chromatographic immunoassay for the qualitative detection of IgG and IgM antibodies (Wuhan UNscience Biotechnology Company UNCOV-40 test kit) following the manufacturer’s guidelines. The test detects the presence of IgM and IgG antibodies directed against the nucleocapsid and the spike proteins of the virus. The manufacturer indicated that the WUHAN test had a clinical sensitivity of 98.511% (95% CI: 96.788%, 99.452%) and specificity of 88.208% (95% CI: 83.086%, 92.221%) (https://www.stratech.co.uk/wp-content/uploads/2020/03/Manual_UNCOV-40_IVD.pdf). One hundred and sixty-eight (23.5%) of the samples were re-run using the nationally recommended Standard-Q COVID-19 IgM/IgG Duo antibody test from SD Biosensor that was reported to have a combined sensitivity of 76.7% (sample sizes = 23/30) (95% CI: 59.1%; 88.2%) and specificity of 98.8% (Sample size = 79/80), (95% CI: 93.3%; 99.8%) https://www.accessdata.fda.gov/cdrh_docs/presentations/maf/maf3274-a001.pdf) for comparison and quality control (QC). The result and image of the cassette were recorded electronically.

### Antibody test comparisons

In Zimbabwe, of the 168 samples rerun on the Standard-Q test, of these samples, 43 had come up positive on the UNCOV-40 test, and 125 had come up negative. The Standard Q test determined fewer positive samples, n = 25; 24 of which had been detected as antibody positive by the UNCOV-40 test. Of the 125 samples denoted negative from the UNCOV-40, the Standard Q test denoted 124 as negative. The summary data is presented in Supplementary Table, S1 Table.

We conducted an independent larger study to compare the sensitivity and specify of 6 different antibody tests in collaboration with colleagues in Ghana as at the time, Zimbabwe did not have a large number of PCR confirmed COVID-19 patients for follow-up (manuscript in preparation). From the results in Ghana we extracted the results for the UNCOV-40 test kit and the Standard Q COVID-19 test. These showed that in 90 PCR confirmed positive SARS-CoV-2 cases and 100 confirmed negative SARS-CoV-2 cases, the specificity of the two tests were closer at 97% for the Standard Q tests and 94% for the UNCOV-40 test, while the sensitivity was higher for UNCOV-40 test at 67% and 48% for the Standard Q (manuscript in preparation).

### RT-PCR diagnosis

In order to diagnose SARS-CoV-2 virus, a nasopharyngeal swab was collected by the local specialist trained COVID-19 Rapid Response Teams set up by the Ministry of Health and Child Care for real time (RT)-PCR following the protocols recommend by the WHO and CDC https://www.who.int/docs/default-source/coronaviruse/whoinhouseassays.pdf. The PCR testing was conducted at currently designated testing sites including the National Tuberculosis Reference Laboratory in Bulawayo and the Lancet Laboratory in Harare. The kits used targeted two separate SARS-COV-2 genes as recommended by the WHO. The laboratory staff amplified the virus nucleocapsid (N) gene and orf1ab genes using the human nuclear RNase P (RNasep) gene as the internal control. PCR positive and negative samples were also run in the assay. PCR analysis were conducted on samples from all participants who were found to be antibody positive or who had reported contact with a suspected or confirmed COVID-19 case. For these samples, PCR analyses were run for each sample. For samples negative for SARS-CoV-2 antibodies, showing no COVID-19 symptoms and with no history of contact with SARS-CoV-2 samples were pooled [11] in groups of 10 as reported from other African countries [12] and analysed. The antibody and PCR results were also entered on electronic forms on the Android devices and descriptive statistics conducted on the data.

The seroprevalence and 95% CIs were calculated and the odds ratios and 95% CIs were also calculated to assess demographic and job characteristics associated with risk of COVID-19 infection.

## Results

### Study uptake

**Fig 2:**
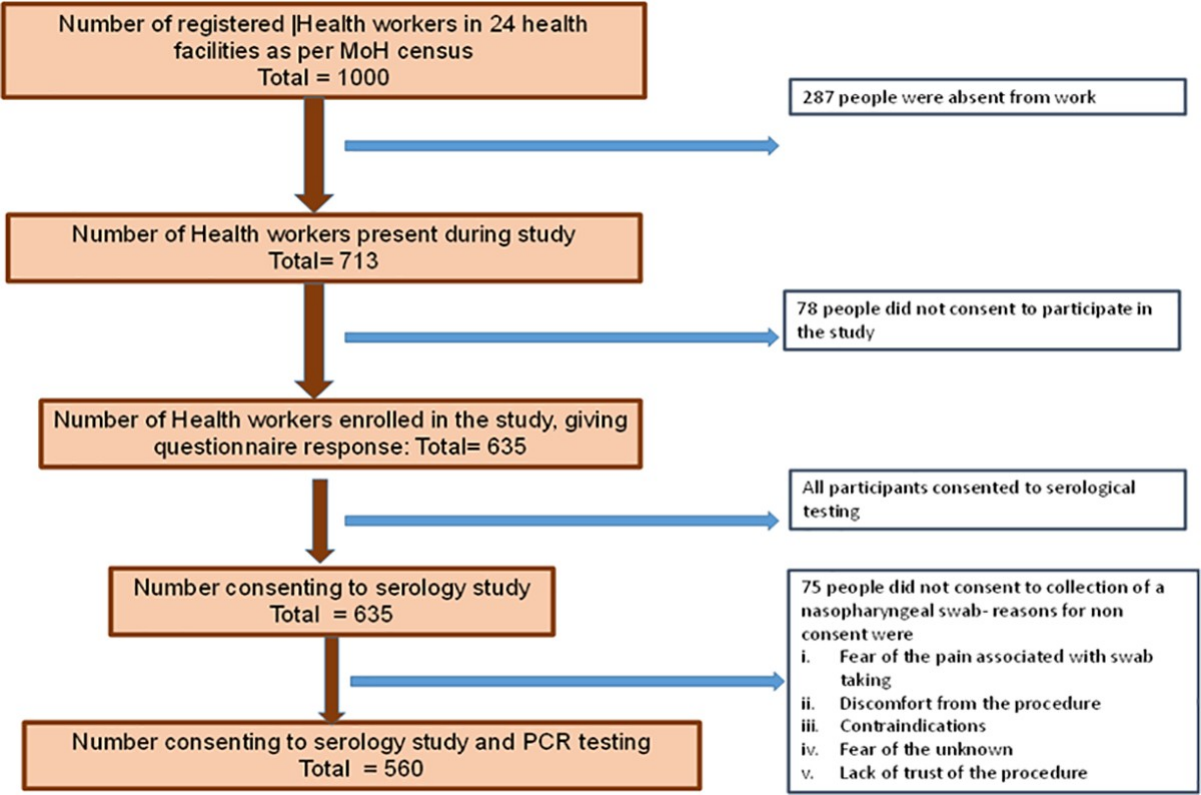
Summary of the study participant sample sizes.

The study invited the 713 patient-facing staff that were present at 24 out of 35 health facilities in Bulawayo at the time of recruitment. The health facilities were partitioned by ownership into those privately owned and those owned by the government and local council (Table 1). The overall uptake rate to the questionnaire and blood samples was 89.1% while the uptake for the nasopharyngeal swabs for the PCR testing was 78.5%. Of the invited 713 people, 635 consented to take part in the study (see Fig 2). Of the 635, 124 (19.5%) reported previous contact with a confirmed COVID-19 case.

**Table 1:** Invited and consenting participants partitioned by type of health facility.

Health facility	Number of participants invited	Questionnaire n (%)	Providing blood samples n (%)	Providing swabs n (%)
Private	269	267(99.3)	267(99.3)	223(82.9)
Municipal	198	168(84.8)	168(84.8)	141(71.2)
Government	246	200(81.3)	200(81.3)	196(79.7)
Overall	713	635(89.1)	635(89.1)	560(78.5)

### Types of patient-facing health workers

**Fig 3:**
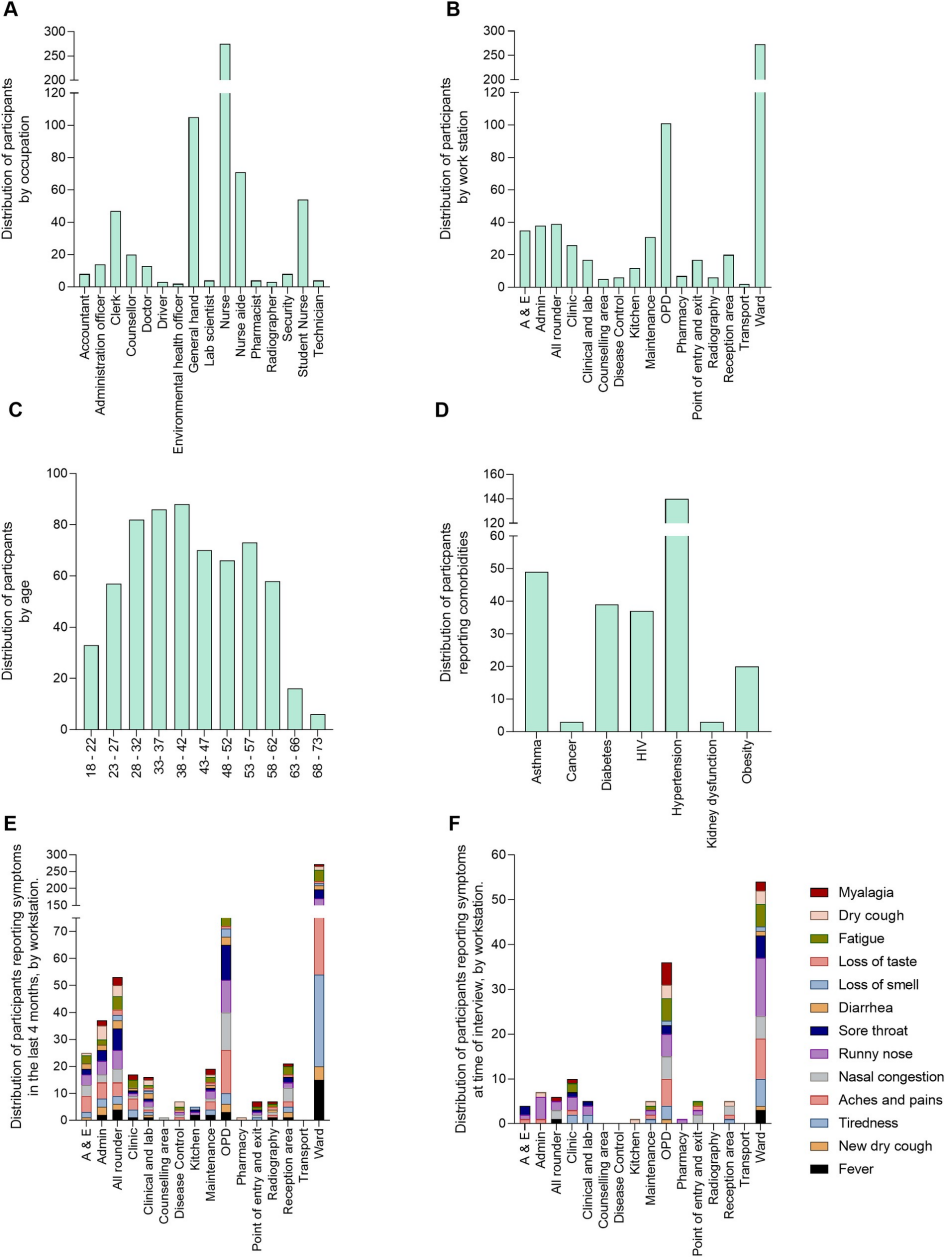
Study population represented as %. A) Partitioned by occupation. B) Partitioned by workstation. C) Partitioned by age group. D) Partitioned by self-reported co-morbidities. E) Partitioned by self-reported COVID-19-like symptoms and work station within the previous 4 months. F) Partitioned by self-reported COVID-19-like symptoms and work station at time or enrolment into the study.

Subsequent analysis focused on the participants who consented to both the questionnaire survey and blood sampling for antibody testing (n = 635). Of these participants, 166 (26.1%) were males and 469(73.9%) were females. Their age ranged from 18 to 73 years with a median (IQR) age of 40.0(32–52) years. Stratifying the study population by occupation and work station indicated that the largest group of patient-facing health workers were nurses who made up 45.3% of the workforce and 43% of the workforce was based in the clinical wards followed by the workforce in the outpatient departments (OPD) at 15.9%. The range of occupations and workstations is shown in Fig 3. Of the 635 participants, over a third (39.1%, n = 248) reported having a history of at least one possible COVID-19 symptom while 14.2% (n = 90) reported at least one current symptom of COVID-19.

**Fig 4:**
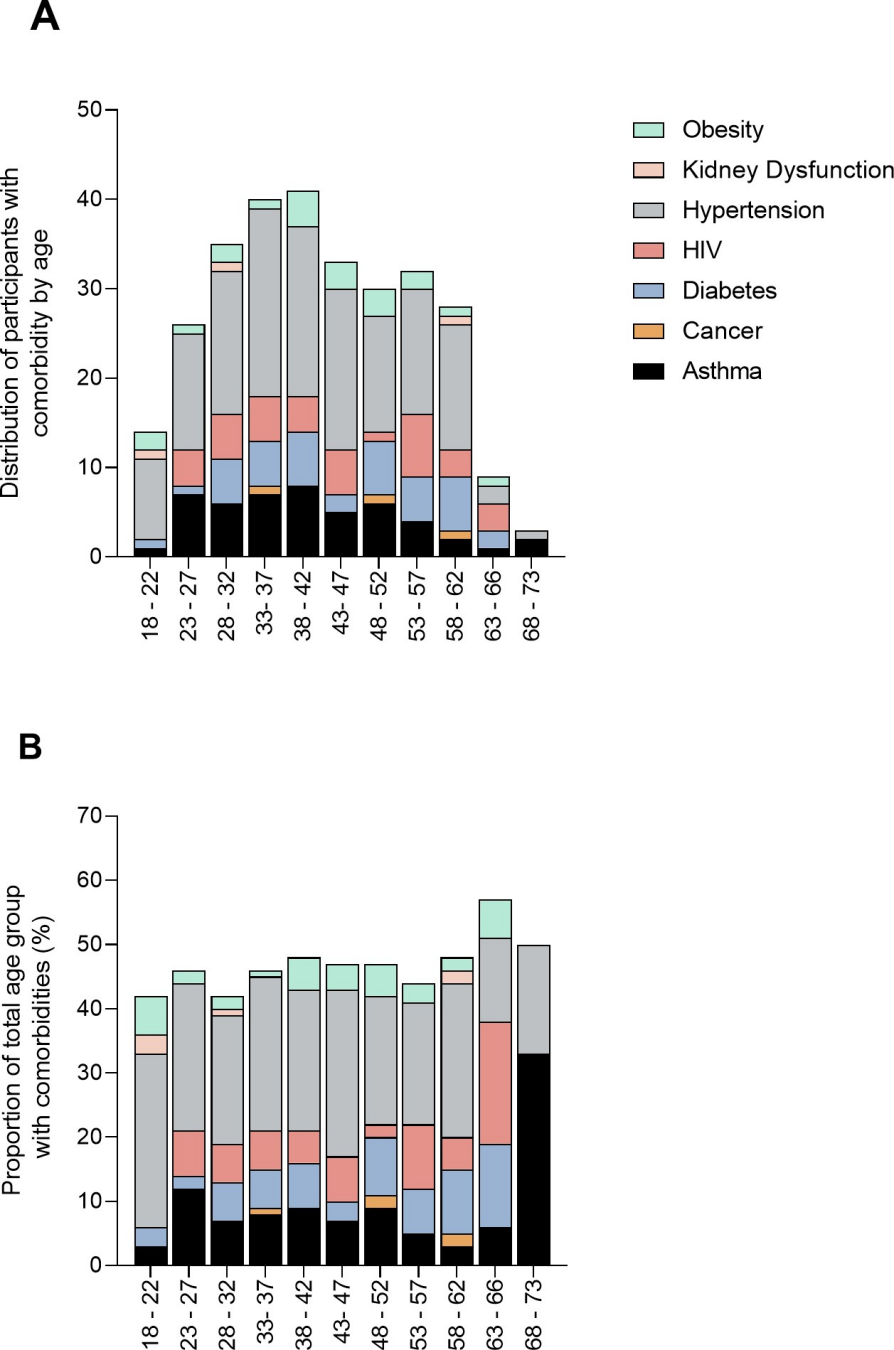
Distribution of the participants partitioned by age and reported co-morbidities as absolute numbers (A) and percentages (B).

We also partitioned the population based on self-reported comorbidities (Fig 4)

### SARS-CoV-2 sero-reactivity and infection status

The presence of IgM and IgG antibodies directed against the SARS-CoV-2 nucleocapsid and the spike proteins was tested in the 635 participants. Among these, 57 people (8.9%: 95% CI = 6.9–11.5) were positive for either IgM or IgG. Of these, 28 people were positive for IgM alone, 26 people were positive for IgG alone and 3 people were positive for both IgM and IgG. Test results obtained using the two different kits were broadly concordant. Relating these results to the sensitivity data for the UNCOV-40 test provided by the manufacturer of 98.5% sensitivity, 1.5% of infections would be missed by the test. This would mean 58 instead of 57 participants were actually seropositive (9.13% instead of 8.9% of the study participants). This is within the 95% confidence interval of the study findings. Using the sensitivity value obtained from Ghana for the same test of 67%, 33% of the positive samples (28 people) would be missed reducing the total from 75 i.e. 13% of the 635 participants.

**Fig 5:**
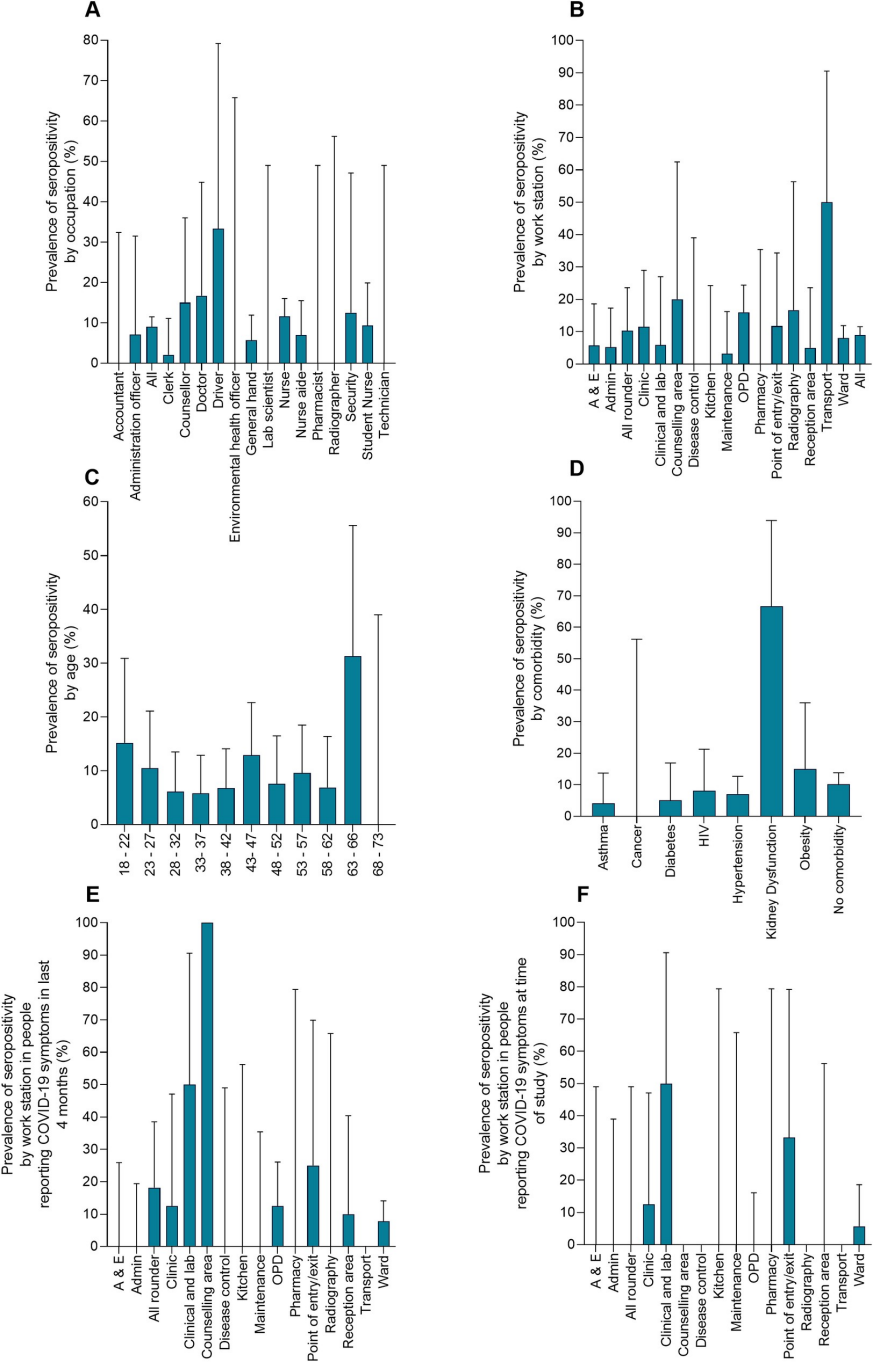
Prevalence of SARS-CoV-2 antibodies in the participating population. A) Partitioned by occupation. B) Partitioned by workstation.C) Partitioned by age group. D) Partitioned by self-reported co-morbidities. E) Partitioned by self-reported COVID-19-like symptoms and work station within the past 4 months. F) Partitioned by self-reported COVID-19-like symptoms during the study period.

**Fig 6:**
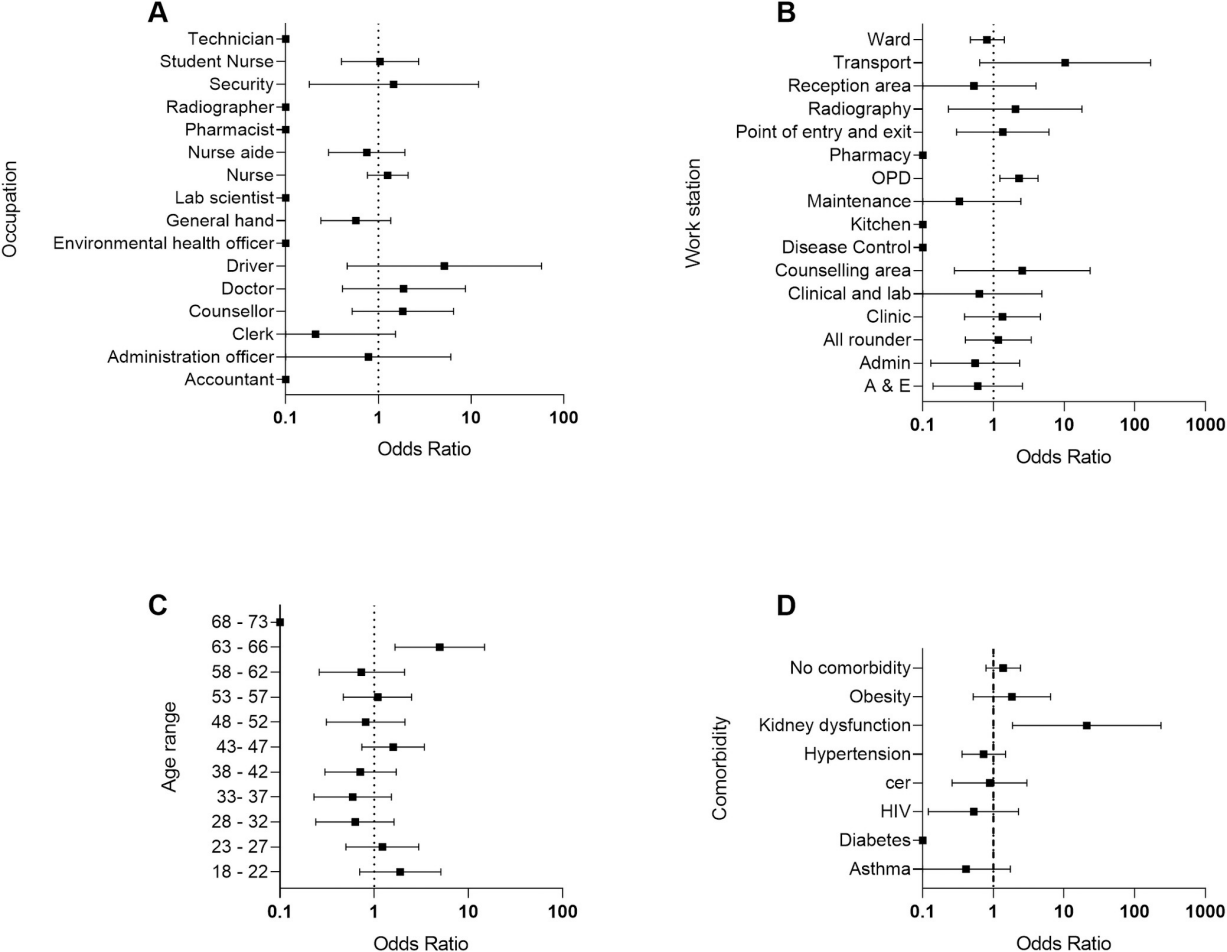
Odds ratio for sero-reactivity. A) Partitioned by occupation. B) Partitioned by workstation. C) Partitioned by age group. D) Partitioned by self-reported co-morbidities.

Of these seropositive people 15.8% (9 people) reported previous contact with a confirmed COVID-19 case. The distribution of the seropositive people by occupation, work station, age and self-reported comorbidities shown in Fig 5. The ward and OPD work stations had a greatest number of seropositive people. Odds ratios indicated that few factors were associated with sero-positivity (Fig 6) (data in Supplementary Tables, S2–S4 Tables). The odds ratios calculations indicated that seropositivity was associated with working in OPD (OR 2.3, 95% CI = 1.23–4.28), aged 63–66 (OR 4.96, 95% CI = 1.66–14.81), years old and having kidney dysfunction as a comorbidity (OR = 20.98, 95% CI = 1.87–235.1).The sample sizes of the different groups are given in Supplementary Tables, S5–S8 Tables. Analysis of the relationship between seropositivity and reported symptoms in the previous 4 months (i.e. since the first case of COVID-19 in Zimbabwe) OR = 1.07 (95% CI = 0.61–1.86) or during our study OR = 0.56 (95% CI = 0.22–1.44), showed no association. When looking at the symptoms that have been most widely associated with COVID-19 i.e. loss of smell (anosmia) or loss of taste (ageusia), none of the antibody positive participants reported anosmia or ageusia at the time of the study. Among the 57 antibody positive participants, 8 reported having at least one symptom, of which 2 reported fever at the time of the study. Of the remaining participants, a total of 19 participants reported either anosmia or ageusia (13 reporting either symptom and 7 reporting both). The analysis of seropositivity and contact with a confirmed with a COVID-19 case also showed no association (OR = 0.98, 95% CI = 0.49–1.96).

Out of the 653 people consenting to antibody testing, 560 also consented to collection of a nasopharyngeal sample and RT-PCR testing to detect the presence of the SARS-CoV-2 infection. Two samples tested positive by PCR giving a prevalence of 0.36%. The two participants who tested positive by PCR were negative for SARS-COV-2 antibody, but were among the 111 participants who had reported contact with a confirmed COVID-19 case.

## Discussion

Health workers are critical for the functioning of a health delivery system and they need to be protected from infection. Furthermore, by preventing nosocomial infections, they form a protective shield between the hospital patients and also between hospital and the communities they live in. It is clear from recent studies that health systems [13] and health workers [14] have been at the forefront of the global response to the SARS-COV-2 pandemic. Therefore, improvements in biosecurity and biosafety have been priorities for infection prevention and control (IPC) as detailed by the WHO [15] for health workers [16]. In response to the health needs of Zimbabwean health workers and in response to the WHO advice on serological tests, we conducted a study to strengthen Zimbabwe’s COVID-19 response focusing on front-line health workers. We characterised the patient-facing health workers in terms of demography and comorbidity risk factors for SARS-CoV-2 infection and disease. It is critical to characterise the different types of occupations where employees come into contact with potential COVID-19 patients to inform IPC within health facilities. This study in privately owned as well as state (government and local council) owned health centres characterised the different groups of patient-facing health workers including nurses, doctors, nurse aides, general hands, administration staff, groundsmen and drivers. These health workers were predominantly stationed in the wards and outpatient departments (OPD). The study also highlighted that the females formed the larger (73.9%) health workforce in our setting.

Several studies have indicated that some comorbidities predisposed to SARS-CoV-2 infection and COVID-19 disease and the list of these was recently updated by the CDC [9]. Given the recent studies highlighting the risk of COVID-19 in black and ethnic minorities in the United Kingdom [17], we also surveyed the participants in this study for self-reported comorbidities that have been listed by the CDC as potential risk factors for SARS-CoV-2 infection and disease. The most prevalent co-morbidity was hypertension (22%), followed by asthma (7%) and diabetes (6%). This trend is similar to that reported in China with hypertension being the most prevalent comorbidity at 16%, followed by diabetes 8.2% [18]. Knowing the levels of co-morbidities in the health workforce is important for informing health messaging to the health workers [19]. In a separate study we have detailed the knowledge, attitudes and practises of these health workers (manuscript in preparation).

The serology study indicated that more participants were willing to have an antibody test than those willing to have a nasopharyngeal swab. The main reasons for the reluctance to have the swab collection procedure were fear of pain and discomfort associated with swab taking based on previous experience and lack of trust of the procedure. Of the participants consenting to antibody testing, 8.9% were seropositive for SARS-CoV-2 antibodies. The sensitivity of the rapid serology test as stipulated by the manufacturer was of 98.5%. This would mean 58 instead of 57 participants were seropositive i.e. 9.13 vs. 8.9% of the study participants. Nonetheless, the value of 9.13 is within the 95% confidence interval of our study findings. Using the sensitivity value obtained from Ghana for the same test of 67% (manuscript in preparation), the seroprevalence from our study would be 13%. Considering differences in our study participants and those used in validating the serology test kit in China and Ghana, there may be difference which influence the seroprevalence we detected. For example, none of the participants in our study had clinically confirmed COVID19 which was the case for both the China and Ghana studies. Furthermore, given that SARS-CoV-2 antibody levels are dynamic and their duration in circulation has not yet been determined in the Zimbabwean population, the time between exposure and antibody detection would be important.

None of the antibody positive participants reported anosmia or ageusia at the time of the study. Among the 57 antibody positive participants, 8 reported having at least one symptom, of which 2 reported fever at the time of the study. Of the remaining participants, a total of 19 participants reported either anosmia or ageusia (13 reporting each and 7 reporting both). The low numbers of people reporting symptoms might be because symptomatic people may have stayed at home as per government guidelines and these people may be among the 287 who did not report to work during our study.

During the study, a total of 64 cases of COVID-19 (out of a total of 512 cumulative cases in the country) had been confirmed in the city and the health centres we surveyed included the designated COVID-19 health centres in the city. None of the seropositive participants were PCR positive for SARS-CoV-2, but the two participants who were PCR positive both reported recent contact with a COVID-19 case. The seroprevalence data and PCR data indicate that levels of exposure to SARS-CoV-2 as well as infection was higher in the health workers than general population. As explained earlier, this is not surprising as they would be more likely to make contacts with infected people due to their occupation, working in the COVID-19 health centres in the country especially returnees from South Africa where levels of infection were higher than in Zimbabwe at the time. In addition, this pattern may have been due to the fact that the study was conducted in the early phase of the epidemic in the country.

We conducted this study during the rising phase of the SARS-CoV-2 epidemic in Zimbabwe. As of 18^th^ September 2020, Zimbabwe had recorded 7647 COVID-19 cases, 5,883 recoveries and 224 deaths. A study of health workers at a tertiary health centre in Belgium reported a seroprevalence of 6.8% in April, 6 weeks after the first case was detected in Belgium [20]. Our study highlighted that kidney dysfunction and age between 63 and 66 years among the health workers were associated with SARS-CoV-2 sero-reactivity. However, our findings are based on small sample sizes of participants testing positive for SARS-CoV-2 antibodies.

There are limitations associated with conducting a seroepidemiology study for an infection with very low prevalence as was the case of SARS-CoV-2 epidemic in Zimbabwe at the time of study. Studies using similar or smaller sample sizes [4] conducted in countries with higher infection prevalences [4] reported about double the seroprevalence in health workers. A seroepidemiology survey post peak of the epidemic will give an indication of the extent of exposure of health workers to infection and disease. Focusing on health workers who were predominantly asymptomatic means details of risk of disease or severe outcomes of COVID-19 were not explored.

Overall, this study highlights that frontline health workers include several non-clinical staff who are patient-facing and that these may also be exposed to SARS-CoV-2 infection during their duty. Although our sample size was too small for detailed analysis to determine differential occupation risk, it is clear from other studies that occupational risk varies across occupation and work stations. For example, a recent study of 545 asymptomatic healthcare workers at University Hospitals Birmingham NHS Foundation Trust (UHBFT) in the UK [4] reported that seroprevalence was highest among housekeepers (34.5%) and those working in acute medicine (33%) or general internal medicine (30.3%), with lower seroprevalence among participants working in intensive care medicine (14.8%). In Zimbabwe participants working in the OPD had a higher risk for seropositivity. The UK study attributed differences in occupational risk of SARS-CoV-2 infection have already been attributed in part, to the distribution of personal protective equipment (PPE) in health settings [4]. In Zimbabwe, the higher risk of seropositivity in the OPD workers may be reflecting the larger number of contacts made with patients since a larger number of health care seekers would visit the OPD compared to those visiting specialist departments.

Prior to our studies there was limited provision of PPE and this was largely in the form of masks and these were mostly targeted at doctors and nurses [21]. Our results highlighted the need for including the various patient-facing health workers in IPC strategies in health centres to ensure effective biosecurity and biosafety. In addition, the study highlighted the need for a complement of PPE and not just face masks. This finding from our study has already impacted on the IPC response of Zimbabwe to the SARS-CoV-2 pandemic in terms of PPE provision for all levels of patient-facing health workers [22].

## Supporting information

S1 Table Comparison of Antibody tests. (DOCX)

S2 Table. A. Occupation as a predictor of serum positivity for SARS-CoV-2 antibodies. B. Work station as a predictor of serum positivity of SARS-CoV-2 antibodies (DOCX)

S3 Table Age as a predictor of serum positivity of SARS-CoV-2 antibodies. (DOCX)

S4 Table Comorbidity as a predictor of serum positivity of SARS-CoV-2 antibodies. (DOCX)

S5 Table Distribution of participants by occupation and seropositivity. (DOCX)

S6 Table Distribution of participants by workstation and seropositivity. (DOCX)

S7 Table Distribution of participants by age range and seropositivity. (DOCX)

S8 Table Distribution of participants by comorbidity and seropositivity. (DOCX)
