# Supplementary material for: SARS-CoV-2 Serological testing in frontline health workers in Zimbabwe
Source: PLoS Negl Trop Dis. 2021 Mar 31;15(3):e0009254. doi: 10.1371/journal.pntd.0009254 (PMC8057594; doi:10.1371/journal.pntd.0009254)
Supplement: S1 Table — (DOCX) [file pntd.0009254.s001.docx]

**Supplementary Tables**

**Table S1: Comparison of Antibody tests**

|  | StandardQ Covid-19 IgM/IgG DuoTest Kit  **POSITIVE** | StandardQ Covid-19 IgM/IgG DuoTest  **NEGATIVE** | TOTAL |
| --- | --- | --- | --- |
| UNSCIENCE UNICOV-40 COVID-19 IgG/IgM Test **POSITIVE** | 24 | 19 | **43** |
| UNSCIENCE UNICOV-40 COVID-19 IgG/IgM Test  **NEGATIVE** | 1 | 124 | **125** |
| **TOTAL** | **25** | **143** | **168** |
