# Supplementary material for: SARS-CoV-2 Serological testing in frontline health workers in Zimbabwe
Source: PLoS Negl Trop Dis. 2021 Mar 31;15(3):e0009254. doi: 10.1371/journal.pntd.0009254 (PMC8057594; doi:10.1371/journal.pntd.0009254)
Supplement: S2 Table. A. Occupation as a predictor of serum positivity for SARS-CoV-2 antibodies. B. Work station as a predictor of serum positivity of SARS-CoV-2 antibodies — (DOCX) [file pntd.0009254.s002.docx]

| **Table S2a: Occupation as a predictor of serum positivity for SARS-CoV-2 antibodies** | | | |
| --- | --- | --- | --- |
|  | No with SARS-CoV-2 antibody in serum positive/negative | |  |
|  | Exposure present | Exposure absent | Odds ratio (95% CI) |
| Accountant | 0/8 | 57/570 | 0 (0-1) |
| Administration officer | 1/13 | 56/565 | 0.78 (0.1-6.04) |
| Clerk | 1/46 | 56/532 | 0.21 (0.03-1.53) |
| Counsellor | 3/17 | 54/561 | 1.83 (0.52 - 6.46) |
| Doctor | 2/11 | 55/567 | 1.87 (0.41-8.67) |
| Driver | 1/2 | 56/576 | 5.14 (0.46 - 57.61) |
| Environmental health officer | 0/2 | 57/576 | 0 (0-1) |
| General hand | 6/99 | 51/479 | 0.57 (0.24-1.36) |
| Lab scientist | 0/4 | 57/574 | 0 (0-1) |
| Nurse | 32/243 | 25/335 | 1.26 (0.76-2.09) |
| Nurse aide | 5/66 | 52/512 | 0.75 (0.29-1.93) |
| Pharmacist | 0/4 | 57/574 | 0 (0-1) |
| Radiographer | 0/3 | 57/575 | 0 (0-1) |
| Security | 1/7 | 56/571 | 1.46 (0.18-12.05) |
| Student Nurse | 5/49 | 52/529 | 1.04 (0.4-2.72) |
| Technician | 0/4 | 57/574 | 0 (0-1) |

| **Table S2b : Work station as a predictor of serum positivity of SARS-CoV-2 antibodies** | | | | |
| --- | --- | --- | --- | --- |
|  | No with SARS-CoV-2 antibody in serum positive/negative | |  |  |
|  | Exposure present | Exposure absent | Odds ratio (95% CI) |  |
| A & E | 2/33 | 55/545 | 0.6 (0.14-2.57) |  |
| Admin | 2/36 | 55/542 | 0.55 (0.13-2.34) |  |
| All rounder | 4/35 | 53/543 | 1.17 (0.4-3.42) |  |
| Clinic | 3/23 | 54/555 | 1.34 (0.39-4.61) |  |
| Clinical, lab | 1/16 | 56/562 | 0.63 (0.08-4.82) |  |
| Counselling area | 1/4 | 56/574 | 2.56 (0.28-23.32) |  |
| Disease Control | 0/6 | 57/566 | 0 (0-1) |  |
| Kitchen | 0/12 | 57/566 | 0.33 (0.04-2.44 |  |
| Maintenance | 1/30 | 56/548 | 0.33 (0.04-2.44) |  |
| OPD | 16/84 | 41/494 | 2.3 (1.23-4.28) |  |
| Pharmacy | 0/7 | 57/571 | 0 (0-1) |  |
| Point of entry, exit | 2/15 | 55/563 | 1.36 (0.3-6.12) |  |
| Radiography | 1/5 | 56/573 | 2.05 (0.23-17.82) |  |
| Reception area | 1/19 | 56/559 | 0.53 (0.07-4) |  |
| Transport | 1/1 | 56/577 | 10.2 (0.64-166.97) |  |
| Ward | 22/252 | 35/326 | 0.81 (0.47-1.42) |  |
