# Supplementary material for: SARS-CoV-2 Serological testing in frontline health workers in Zimbabwe
Source: PLoS Negl Trop Dis. 2021 Mar 31;15(3):e0009254. doi: 10.1371/journal.pntd.0009254 (PMC8057594; doi:10.1371/journal.pntd.0009254)
Supplement: S3 Table — (DOCX) [file pntd.0009254.s003.docx]

| **Table S3: Age as a predictor of serum positivity of SARS-CoV-2 antibodies** | | | | |
| --- | --- | --- | --- | --- |
|  | No with SARS-CoV-2 antibody in serum positive/negative | |  |  |
|  | Exposure present | Exposure absent | Odds ratio (95% CI) |  |
| 18 - 22 | 5/28 | 52/550 | 1.89 (0.7-5.1) |  |
| 23 - 27 | 6/51 | 51/527 | 1.22 (0.5-2.97) |  |
| 28 - 32 | 5/77 | 52/501 | 0.63 (0.24-1.62) |  |
| 33- 37 | 5/81 | 52/497 | 0.59 (0.23-1.52) |  |
| 38 - 42 | 6/82 | 51/496 | 0.71 (0.3-1.71) |  |
| 43- 47 | 9/61 | 48/517 | 1.59 (0.74-3.4) |  |
| 48 - 52 | 5/61 | 52/517 | 0.81 (0.31-2.12) |  |
| 53 - 57 | 7/66 | 50/512 | 1.09 (0.47-2.49) |  |
| 58 - 62 | 4/54 | 53/524 | 0.73 (0.26-2.1) |  |
| 63 - 66 | 5/11 | 52/567 | 4.96 (1.66 - 14.81) |  |
| 68 - 73 | 0/6 | 57/572 | 0 (0-1) |  |
