# Supplementary material for: SARS-CoV-2 Serological testing in frontline health workers in Zimbabwe
Source: PLoS Negl Trop Dis. 2021 Mar 31;15(3):e0009254. doi: 10.1371/journal.pntd.0009254 (PMC8057594; doi:10.1371/journal.pntd.0009254)
Supplement: S4 Table — (DOCX) [file pntd.0009254.s004.docx]

| **Table S4: Comorbidity as a predictor of serum positivity of SARS-CoV-2 antibodies** | | | | |
| --- | --- | --- | --- | --- |
|  | No with SARS-CoV-2 antibody in serum positive/negative | |  |  |
|  | Exposure present | Exposure absent | Odds ratio (95% CI) |  |
| Asthma | 2/47 | 55/531 | 0.41 (0.1-1.74) |  |
| Cancer | 0/3 | 54/578 | 0 (0-1) |  |
| Diabetes | 2/37 | 55/541 | 0.53 (0.12-2.27) |  |
| HIV | 3/34 | 54/544 | 0.89 (0.26-2.99) |  |
| Hypertension | 10/130 | 47/448 | 0.73 (0.36-1.49) |  |
| Kidney dysfunction | 2/1 | 55/577 | 20.98 (1.87-235.1) |  |
| Obesity | 3/17 | 54/561 | 1.83 (0.52-6.46) |  |
| No comorbidity | 35/309 | 22/269 | 1.38 (0.79-2.42) |  |
