# Supplementary material for: SARS-CoV-2 Serological testing in frontline health workers in Zimbabwe
Source: PLoS Negl Trop Dis. 2021 Mar 31;15(3):e0009254. doi: 10.1371/journal.pntd.0009254 (PMC8057594; doi:10.1371/journal.pntd.0009254)
Supplement: S5 Table — (DOCX) [file pntd.0009254.s005.docx]

| **Table S5: Distribution of participants by occupation and seropositivity** | | | | |
| --- | --- | --- | --- | --- |
| **Occupation** | **Total number** | **Proportion total sample (%)** | **Total seropositive** | **Proportion seropositive (%)** |
| Accountant | 8 | 1.3% | 0 | 0.0% |
| Administration officer | 14 | 2.2% | 1 | 7.1% |
| Clerk | 47 | 7.4% | 1 | 2.1% |
| Counsellor | 20 | 3.1% | 3 | 15.0% |
| Doctor | 13 | 2.0% | 2 | 15.4% |
| Driver | 3 | 0.5% | 1 | 33.3% |
| Environmental health officer | 2 | 0.3% | 0 | 0.0% |
| General hand | 105 | 16.5% | 6 | 5.7% |
| Lab scientist | 4 | 0.6% | 0 | 0.0% |
| Nurse | 275 | 43.3% | 32 | 11.6% |
| Nurse aide | 71 | 11.2% | 5 | 7.0% |
| Pharmacist | 4 | 0.6% | 0 | 0.0% |
| Radiographer | 3 | 0.5% | 0 | 0.0% |
| Security | 8 | 1.3% | 1 | 12.5% |
| Student Nurse | 54 | 8.5% | 5 | 9.3% |
| Technician | 4 | 0.6% | 0 | 0.0% |
| All | 635 | 100.0% | 57 | 9.0% |
