# Supplementary material for: SARS-CoV-2 Serological testing in frontline health workers in Zimbabwe
Source: PLoS Negl Trop Dis. 2021 Mar 31;15(3):e0009254. doi: 10.1371/journal.pntd.0009254 (PMC8057594; doi:10.1371/journal.pntd.0009254)
Supplement: S6 Table — (DOCX) [file pntd.0009254.s006.docx]

| **Table S6: Distribution of participants by workstation and seropositivity** | | | | |
| --- | --- | --- | --- | --- |
| **Work station** | **Total number** | **Proportion total sample (%)** | **Total seropositive** | **Proportion seropositive (%)** |
| A & E | 35 | 5.5% | 2 | 5.7% |
| Admin | 38 | 6.0% | 2 | 5.3% |
| All rounder | 39 | 6.1% | 4 | 10.3% |
| Clinic | 26 | 4.1% | 3 | 11.5% |
| Clinical and lab | 17 | 2.7% | 1 | 5.9% |
| Counselling area | 5 | 0.8% | 1 | 20.0% |
| Disease Control | 6 | 0.9% | 0 | 0.0% |
| Kitchen | 12 | 1.9% | 0 | 0.0% |
| Maintenance | 31 | 4.9% | 1 | 3.2% |
| OPD | 101 | 15.9% | 16 | 15.8% |
| Pharmacy | 7 | 1.1% | 0 | 0.0% |
| Point of entry and exit | 17 | 2.7% | 2 | 11.8% |
| Radiography | 6 | 0.9% | 1 | 16.7% |
| Reception area | 20 | 3.1% | 1 | 5.0% |
| Transport | 2 | 0.3% | 1 | 50.0% |
| Ward | 273 | 43.0% | 22 | 8.1% |
| All | 635 | 100.0% | 57 | 9.0% |
