# Supplementary material for: SARS-CoV-2 Serological testing in frontline health workers in Zimbabwe
Source: PLoS Negl Trop Dis. 2021 Mar 31;15(3):e0009254. doi: 10.1371/journal.pntd.0009254 (PMC8057594; doi:10.1371/journal.pntd.0009254)
Supplement: S7 Table — (DOCX) [file pntd.0009254.s007.docx]

| **Table S7: Distribution of participants by age range and seropositivity** | | | | | |
| --- | --- | --- | --- | --- | --- |
| **Age Range** | **Total number** | **Proportion total sample (%)** | **Total seropositive** | **Proportion seropositive (%)** |  |
| 18 - 22 | 33 | 5.2% | 5 | 15.2% |  |
| 23 - 27 | 57 | 9.0% | 6 | 10.5% |  |
| 28 - 32 | 82 | 12.9% | 5 | 6.1% |  |
| 33- 37 | 86 | 13.5% | 5 | 5.8% |  |
| 38 - 42 | 88 | 13.9% | 6 | 6.8% |  |
| 43- 47 | 70 | 11.0% | 9 | 12.9% |  |
| 48 - 52 | 66 | 10.4% | 5 | 7.6% |  |
| 53 - 57 | 73 | 11.5% | 7 | 9.6% |  |
| 58 - 62 | 58 | 9.1% | 4 | 6.9% |  |
| 63 - 66 | 16 | 2.5% | 5 | 31.3% |  |
| 68 - 73 | 6 | 0.9% | 0 | 0.0% |  |
