# Supplementary material for: SARS-CoV-2 Serological testing in frontline health workers in Zimbabwe
Source: PLoS Negl Trop Dis. 2021 Mar 31;15(3):e0009254. doi: 10.1371/journal.pntd.0009254 (PMC8057594; doi:10.1371/journal.pntd.0009254)
Supplement: S8 Table — (DOCX) [file pntd.0009254.s008.docx]

| **Table S8: Distribution of participants by comorbidity and seropositivity** | | | | |
| --- | --- | --- | --- | --- |
| **Comorbidity** | **Total number** | **Proportion total sample (%)** | **Total seropositive** | **Proportion seropositive (%)** |
| Asthma | 49 | 7.7% | 2 | 4.1% |
| Cancer | 3 | 0.5% | 0 | 0.0% |
| Diabetes | 39 | 6.1% | 2 | 5.1% |
| HIV | 37 | 5.8% | 3 | 8.1% |
| Hypertension | 140 | 22.0% | 10 | 7.1% |
| Kidney dysfunction | 3 | 0.5% | 2 | 66.7% |
| Obesity | 20 | 3.1% | 3 | 15.0% |
| No comorbidity | 344 | 54.2% | 35 | 10.2% |
